# Increasing age of patients admitted to intensive care, and association between increased age and greater risk of post-ICU death

**DOI:** 10.1186/cc13246

**Published:** 2014-03-17

**Authors:** B Creagh-Brown, S Green

**Affiliations:** 1 Royal Surrey County Hospital, Guildford, UK

## Introduction

The population of the UK is ageing, with the fastest increase in those ≥85 years. Increased age has been repeatedly associated with adverse outcome and it is uncertain to what extent this relates to the changes of ageing in themselves, or due to other considerations. Age is a key variable in the majority of scoring systems that relate patient characteristics to adverse outcome. We aimed to assess change in age distribution of patients admitted to our ICU over 20 years and examine the relationship between age of patient, mortality and length of stay (LOS).

## Methods

Data were extracted from electronic records (WardWatcher) and analysed using SPSS 20, GraphPad Prism 5.0 and Excel 2007.

## Results

**Figure 1:**
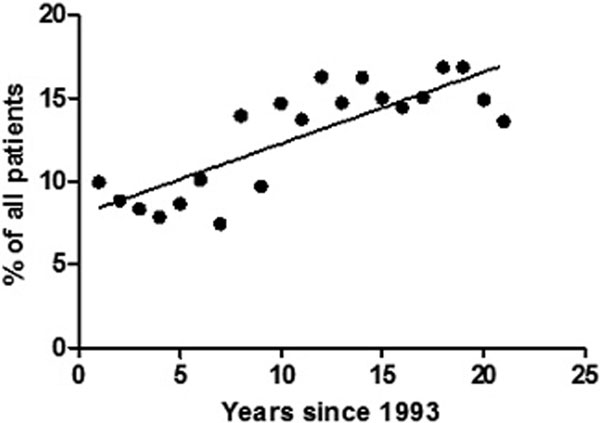
**Proportion of patients admitted to ICU aged >80 years, over time**.

**Figure 2:**
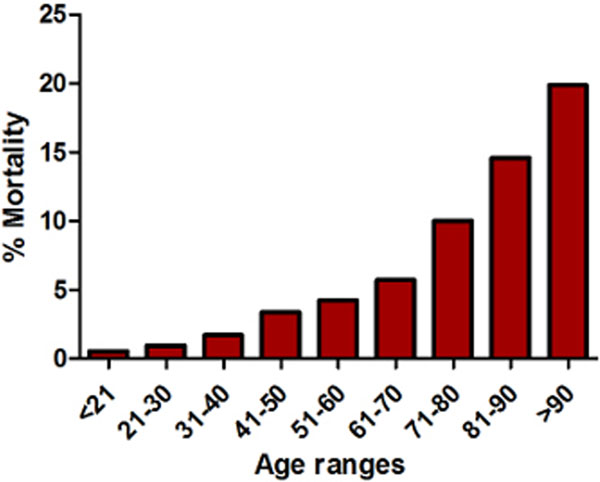
**Risk of long stay versus 1-year mortality**.

ICU patients have become older by 4.4 months/year. By 2013 the median age was 66 and 15% of all patients are now ≥80 years - a 36% increase since 1993. Compared with the reference group (61 to 70 years), those in the older deciles have increased risk of ICU and hospital mortality (*P *< 0.01). Fifteen per cent of all those 81 to 90 years old and 20% of those >91 years old who do not die on the ICU go on to die on the ward. It is unknown what proportion of these post-ICU deaths was unexpected. Older patients had prolonged hospital LOS (*P *< 0.01) but not ICU LOS. See Figures 1 and 2.

## Conclusion

There are increasing numbers of older patients on ICUs in the UK. In analyses uncorrected for severity of illness or comorbidities, older patients are more likely to die on the ICU, and on the ward after ICU. They also spend longer in hospital prior to discharge.

